# Levels of Fibrin Degradation Products at Admission With Acute Ischemic Stroke Correlate With the NIH Stroke Scale Score 1 h After Intravenous Thrombolysis

**DOI:** 10.3389/fneur.2021.651867

**Published:** 2021-05-28

**Authors:** Bin Zhu, Limin Zhang, Wanliang Du, Jie Yang, Yue Tian, Mingfen Wu, Tingxi Wu, Xi Ling, Yilin Liu, Xingquan Zhao, Zhigang Zhao

**Affiliations:** ^1^Department of Pharmacy, Beijing Tiantan Hospital, Capital Medical University, Beijing, China; ^2^Department of Clinical Laboratory, Beijing Tiantan Hospital, Capital Medical University, Beijing, China; ^3^Department of Neurology, Beijing Tiantan Hospital, Capital Medical University, Beijing, China; ^4^State Key Laboratory of Natural Medicines, Research Center of Biostatistics and Computational Pharmacy, China Pharmaceutical University, Nanjing, China

**Keywords:** acute ischemic stroke, NIHSS score, thrombolysis effect, fibrin degradation products, r-tPA

## Abstract

**Background:** Fibrin degradation products (FDPs) are fragments released by the plasmin-mediated degradation of fibrinogen or fibrin. Whether plasma levels of these fragments can predict the thrombolytic effect of recombinant tissue plasminogen activator (r-tPA) remains unknown.

**Methods:** We performed a hospital-based study of patients with acute ischemic stroke (AIS) to explore the relationship between FDP levels at admission and the NIH Stroke Scale (NIHSS) score 1 h after thrombolysis treatment. In this retrospective, single-center study, the data of all patients with AIS who received r-tPA treatment at Beijing Tiantan Hospital from January 2019 to October 2020 were collected and analyzed. Demographic and clinical data, including laboratory examinations, were also analyzed.

**Results:** A total of 339 patients with AIS were included in this study. Of these, 151 showed favorable effects of r-tPA, and 188 showed unsatisfactory effects at 1 h after thrombolysis. Overall, we found an inverse relationship between the FDPs levels at admission and the NIHSS score. A significant difference was observed when using the interquartile range of the FDPs levels (1.31 μg/mL) as a cutoff value (*P* = 0.003, odds ratio [OR] = 1.95, 95% confidence interval [CI]: 1.26–3.01), even after adjusting for confounding factors (*P* = 0.003, OR = 2.23, 95% CI: 1.31–3.77). In addition, significant associations were observed in the tertile (T3) and quartile (Q3, Q4) FDP levels when compared with T1 or Q1. A nomogram was also employed to create a model to predict an unsatisfactory effect of r-tPA. We found that FDP levels, white blood cell count, age, D-dimer level, and body mass index could influence the thrombolytic effect of r-tPA.

**Conclusion:** In conclusion, the present study demonstrated that the levels of FDPs at admission can be used as a prognostic factor to predict the curative effect of r-tPA.

## Introduction

Acute ischemic stroke (AIS) is a major cause of mortality and morbidity worldwide (1, 2). A time-sensitive medical emergency, it is estimated to result in the death of ~1.9 million neurons per minute without treatment (3). Intravenous thrombolysis with recombinant tissue plasminogen activator (r-tPA) is an effective treatment for patients with AIS (4, 5). Timely intravenous r-tPA (alteplase) administered in the early period (≤4.5 h) is recognized as a mainstay of the AIS treatment strategy (4, 6, 7). However, r-tPA has several shortcomings, such as a low recanalization rate, a risk of intracranial hemorrhage, and a short half-life requiring continuous infusion. Therefore, it is vital that the attending physician be able to make a quick assessment and prediction of outcome under tight time constraints (8, 9).

Fibrin degradation products (FDPs), which can compete with fibrinogen for binding to the platelet membrane and thus interfere with platelet aggregation, are fragments released by the plasmin-mediated degradation of fibrinogen or fibrin (10). The FDPs level is very sensitive to intravascular thrombus and may be markedly elevated once the coagulation and fibrinolytic system is activated (11). In most instances, FDP is correlated with inflammation, disseminated intravascular coagulation, acute aortic dissection, pulmonary embolus, and trauma (12, 13). However, its relationship with stroke remains unclear, especially in patients receiving r-tPA. Thus, in this study, we investigated in patients with AIS the association of the FDP level with the curative effect of early r-tPA administration as assessed by the NIH Stroke Scale (NIHSS) score, to support clinicians in making an overall evaluation.

## Methods

### Study Design and Patients

We performed a single-center retrospective case series of patients who received r-tPA for the treatment of AIS between January 2019 and October 2020 at Beijing Tiantan Hospital, China. All patients were admitted within 4.5 h of onset and were diagnosed as having AIS by magnetic resonance imaging or computed tomography, and received an intravenous r-tPA infusion of 0.9 mg/kg (to a maximum of 90 mg) at the Neurology emergency department. Patients that underwent both intravenous thrombolysis and mechanical thrombectomy, or those with large vessel occlusion were excluded. The study was approved by the Ethics Committee of Beijing Tiantan Hospital and conducted in accordance with the Declaration of Helsinki. Due to the retrospective nature of the study, the requirement of written informed consent was waived. Confidential patient information was deleted from the entire data set prior to analysis.

### Data Collection

The laboratory values of coagulative function, including FDP level at first admission (before r-tPA use), were employed. Other medical records, including demographic data, baseline clinical parameters, clinical diagnoses, and therapeutic schedules, were carefully extracted using a standardized case-report form. If any information was unclear, the doctors or other healthcare providers who had been in charge were consulted.

### Statistical Analysis

Patients were classified into favorable- and unfavorable-effect groups using the NIHSS score at admission and at 1 h after r-tPA injection. An NIHSS score of ≤1 point 1 h after thrombolytic therapy or an NIHSS score 1 h after treatment at least 4 points below the score at admission was considered a good thrombolytic effect, while a 1 h decrease in the NIHSS score of <4 points was considered an unfavorable effect (14). Data are presented as medians with interquartile ranges (IQRs) or as means with standard deviations (SDs), while categorical variables were presented as frequencies and percentages (%). Logistic regression analyses were performed to evaluate the relationship between levels of FDPs and the NIHSS score and the risk factors such as age, sex, body mass index (BMI) and so on were adjusted. Results are expressed as adjusted odds ratios (ORs) with 95% confidence intervals (CIs). A nomogram was constructed based on the outcomes of the multivariate analysis. All statistical tests were 2-sided and *P*-values <0.05 were considered to indicate statistical significance. Statistical analyses were performed using Empower Stats (http://www.empowerstats.com) and R software, version 3.3.1 (http://www.R-project.org/).

## Results

### Clinical Characteristics of Patients

**Figure 1:**
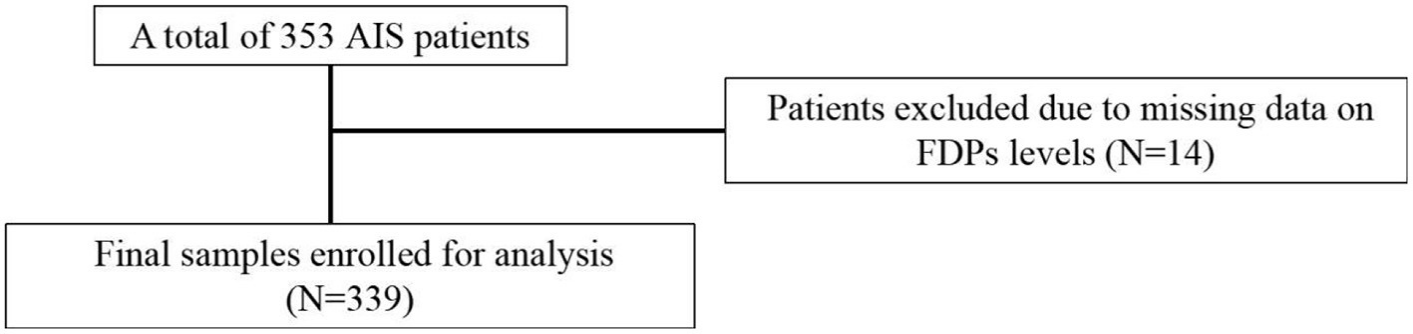
Flow chart of patients for analysis.

A total of 339 patients with AIS were included in this study (Figure 1). Of these, 151 patients (*n* = 105 men) with an average age of 62.6 ± 12.0 y comprised the favorable-effect group and 188 patients (*n* = 145 men) with an average age 62.7 ± 11.4 y comprised the unfavorable-effect group. At admission, the average NIHSS for patients with a favorable effect was 4.0 (2.0, 9.0) and for those with an unfavorable effect, 5.0 (4.0, 9.0). One hour after receiving rt-PA, the NIHSS scores were 1.0 (0.0, 2.0), and 4.0 (2.0, 9.0) for the favorable- and unfavorable-effect groups, respectively. At admission, no significant differences were observed between the two groups in white blood cell counts, lymphocytes, neutrophils, monocytes, C-reactive protein, or prothrombin time (P > 0.05). However, significant intergroup differences were found in FDPs, D-dimer, and the activated partial thromboplastin time (*P* < 0.05) (Table 1).

**Table 1:** Baseline characteristics of the patients enrolled in the study.

**Variables**	**Outcome**		
	**Favorable prognosis**	**Unfavorable prognosis**	***P*-value**
No.	151	188	
Age (y)^*^	62.6 ± 12.0	62.7 ± 11.4	0.980
BMI (kg/m^2^)^*^	25.1 ± 3.4	25.4 ± 3.9	0.412
Gender, *n* (%)^*^			0.146
Male	105 (69.5)	145 (77.1)	
Female	46 (30.5)	43 (22.9)	
Hypertension, *n* (%)^*^			0.695
No	109 (75.2)	145 (78.8)	
Yes	36 (24.8)	39 (21.2)	
Diabetes, *n* (%)^*^			0.286
No	101 (67.3)	137 (73.3)	
Yes	49 (32.7)	50 (26.7)	
Hyperlipemia, *n* (%)^*^			0.517
No	109 (75.2)	145 (78.8)	
Yes	36 (24.8)	39 (21.2)	
Smoking status, *n* (%)^*^			**0.014**
Never	73 (48.3)	65 (34.6)	
Ever	78 (51.7)	123 (65.4)	
Drinking status, *n* (%)^*^			0.557
Never	76 (50.3)	87 (46.5)	
Ever	75 (49.7)	100 (53.5)	
NIHSS score at admission^†^	4.0 (2.0, 9.0)	5.0 (4.0, 9.0)	**0.003**
NIHSS score after rt-PA 1 hour^†^	1.0 (0.0, 2.0)	4.0 (2.0, 9.0)	**<0.001**
ASPECT score at admission^†^	10 (8, 12)	9 (8, 11)	**0.027**
WBC (×10^9^/L)^†^	7.3 (6.2, 8.9)	7.6 (6.1, 9.3)	0.272
LY (×10^9^/L)^†^	1.7 (1.3, 2.3)	1.6 (1.2, 2.1)	0.111
NEUT (×10^9^/L)^†^	4.5 (3.6, 6.5)	5.1 (4.0, 6.7)	**0.036**
MONO (×10^9^/L)^†^	0.4 (0.3, 0.5)	0.4 (0.3, 0.5)	0.485
D-D (ug/ml)^†^	0.6 (0.4, 0.8)	0.6 (0.4, 0.9)	**0.044**
FDP (ug/ml)^†^	1.2 (0.9, 1.8)	1.5 (1.0, 2.1)	**0.004**
PT(INR)^†^	1.0 (1.0, 1.0)	1.0 (1.0, 1.1)	0.518
APTT time (second)^†^	29.8 (28.2, 31.6)	28.9 (26.7, 31.1)	**0.009**
CRP (mg/L)^†^	1.5 (0.7, 3.2)	1.6 (0.7, 3.3)	0.747

*BMI indicates body mass index; FDP indicates fibrin degradation products; WBC indicates white blood counts; TIA indicates transient ischemic attack*;

* *For continuous variables, values are presented as mean ± SD*.

† *Values are presented as median (IQR); Significant associations are marked with bold*.

### Relationship of the FDP Level to the r-tPA Thrombolysis Effect

**Figure 2:**
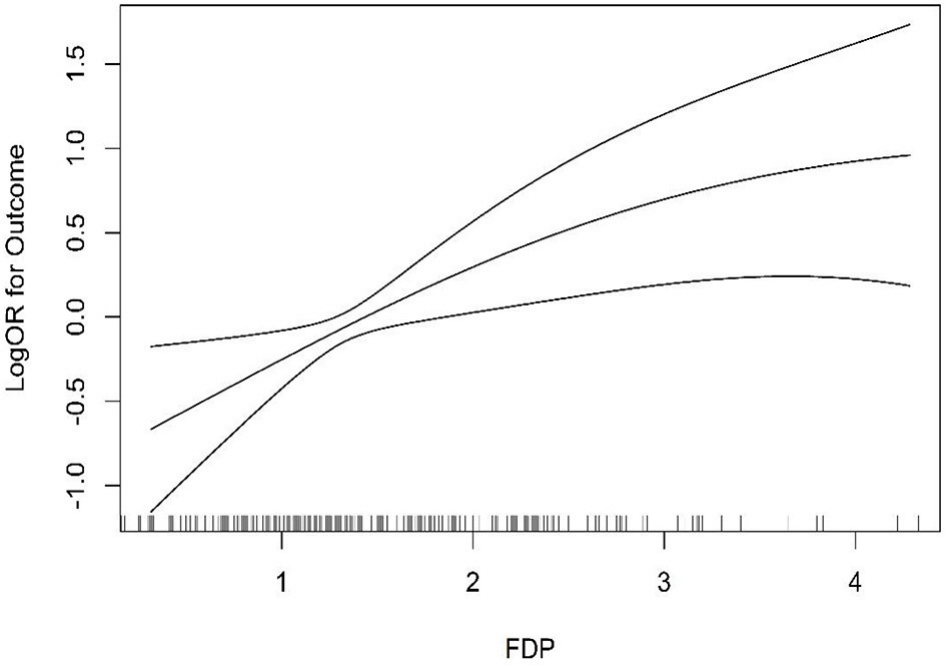
Smooth curves between FDP level at admission and thromblysis outcome.

At first, we found no significant association (*P* = 0.306, OR = 1.06, 95% CI = 0.95–1.17) even after adjusting for confounding factors such as gender, age, and BMI (*P* = 0.068, OR = 1.18, 95% CI = 0.99–1.40). However, to further explore the influence of FDP levels on the r-tPA thrombolysis effect, the second quartile, tertiles, and higher quartiles of the FDP levels were used for further logistic regression analysis (Table 2). When using the FDP level (1.31 ug/mL) as a cutoff value, we found a significant association between FDP levels and the thrombolysis effect (*P* = 0.003, OR = 1.95, 95% CI: 1.26–3.01), even after adjusting for confounding factors (*P* = 0.003, OR = 2.23, 95% CI: 1.31–3.77). The tertile data cutoffs were T1 < 1.09, T2 = 1.09–1.72, and T3 > 1.72; compared with T1, we found that patients with FDP levels in T3 showed a correlation with the r-tPA thrombolysis effect (*P* = 0.008, OR = 2.06, 95% CI: 1.21–3.51; adjusting for confounding factors, *P* = 0.003, OR = 2.70, and 95% CI: 1.39–5.26). In addition, compared with Q1, we found significant differences in the third quartile (*P* = 0.014, OR: 2.14, 95 % CI: 1.17–3.91) and fourth quartile (*P* = 0.014, OR: 2.21, 95% CI: 1.18–4.15), even after adjusting for confounding factors (Q3, *P* = 0.013, OR = 2.47, 95% CI: 1.21–5.02; Q4, *P* = 0.007, OR = 3.00, 95% CI: 1.36–6.65) (Figure 2).

**Table 2:** The logistic regression analysis of the association between intravenous thromblysis outcomes of h after r-tPA and FDP level at admission.

	***N***	**Case (%)**	**Crude model**	***P-*value**	Adjusted model^*^	***P-value***
			**OR (95%CI)**		**OR (95%CI)**	
FDP, ug/ml	339	188 (55.5)	1.06 (0.95, 1.17)	0.306	1.18 (0.99, 1.40)	0.068
Median						
B1 (<1.31)	171	81 (47.4)	*ref*		*ref*	
B2 (≥1.31)	168	107 (63.7)	1.95 (1.26, 3.01)	**0.003**	2.23 (1.31, 3.77)	**0.003**
Tertiles						
T1 (<1.09)	113	52 (46.0)	*ref*		*ref*	
T2 (1.09–1.72)	113	64 (56.6)	1.53 (0.91, 2.59)	0.111	1.79 (0.98, 3.27)	0.060
T3 (≥1.72)	113	72 (63.7)	2.06 (1.21, 3.51)	**0.008**	2.70 (1.39, 5.26)	**0.003**
Quartiles						
Q1 (<0.95)	85	38 (44.7)	*Ref*		*ref*	
Q2 (0.95–1.31)	86	43 (50.0)	1.24 (0.68, 2.26)	0.488	1.38 (0.70, 2.75)	0.352
Q3 (1.31–1.98)	90	57 (63.3)	2.14 (1.17, 3.91)	**0.014**	2.47 (1.21, 5.02)	**0.013**
Q4 (≥1.98)	78	50 (64.1)	2.21 (1.18,4.15)	**0.014**	3.00 (1.36, 6.65)	**0.007**

* *Adjusted for gender, age, BMI, hypertension, diabetes, hyperlipemia, smoking status and drinking status. Significant associations are marked with bold*.

### A Predictive Model for the r-tPA Thrombolysis Effect

**Figure 3:**
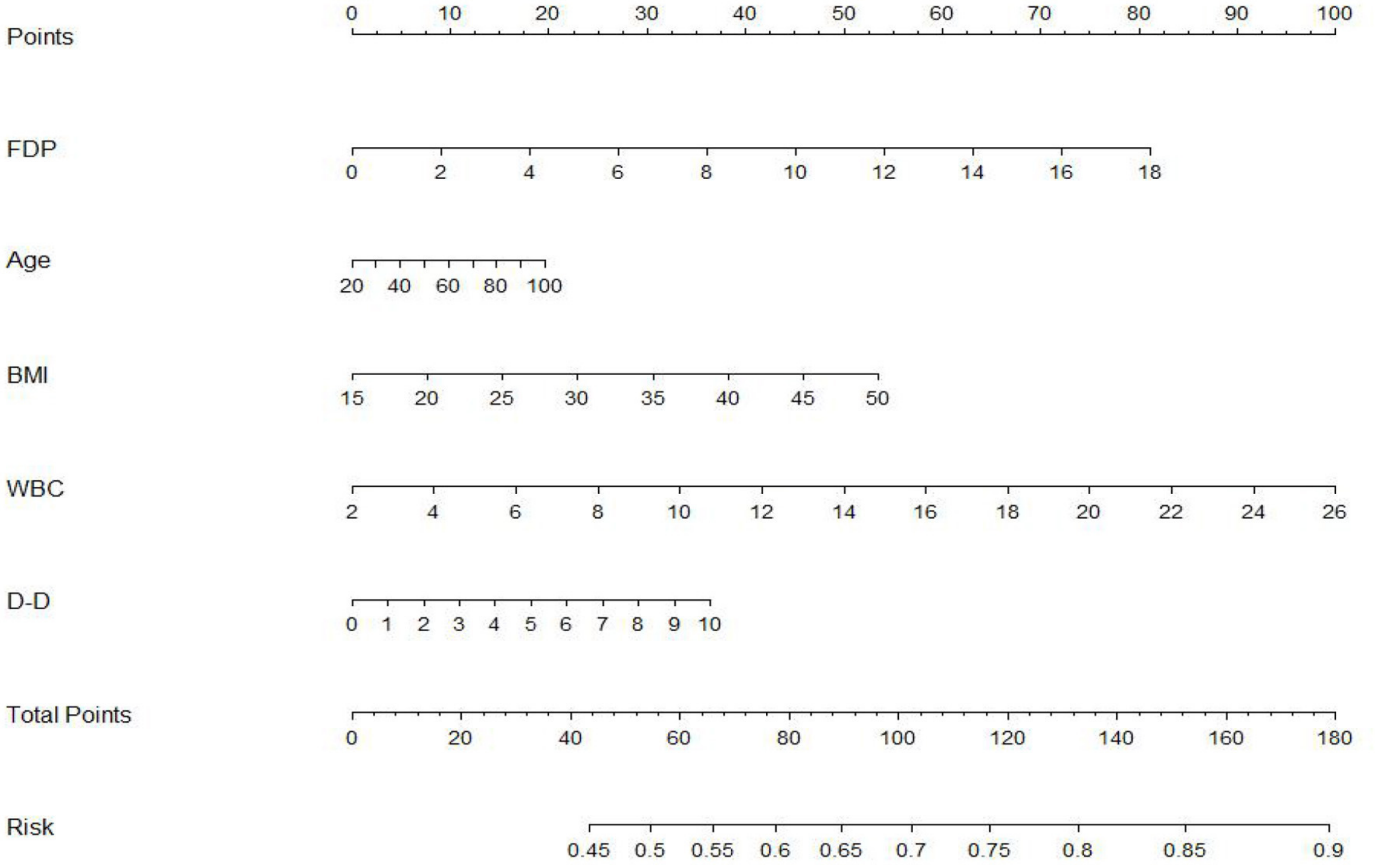
The nomogram developed by logistic regression. Each selected variable is represented by a line in the figure. According to the value, each variable receives a point. Total points are added for each variable and matched with the probability of bad outcome.

To further evaluate the predictive value of FDP levels with thrombolysis effect in patients with AIS, a non-invasive nomogram was developed using data for all patients based on five parameters identified by the logistic regression analysis (Figure 3). It is predicted that a higher total nomogram score will be associated with a higher likelihood of a poor thrombolytic effect.

## Discussion

Intravenous r-tPA, which has been a standard treatment for acute ischemic stokes for 25 years (15), converts the plasminogen to the proteolytic enzyme plasmin, which lyses fibrin as well as fibrinogen (16). Applying rt-PA to achieve intravenous thrombolysis within 4.5 h has been proven beneficial for treating patients with ischemic stroke. However, there are reports indicating that while there was no effect on some patients, some showed deterioration in clinical manifestations (such as symptomatic intracranial hemorrhage) in response to rt-PA (17, 18). AIS treatment is time sensitive, requiring rapid assessment and decision making. Thrombolysis within 4.5 h of onset of AIS has shown to improve its outcomes by reducing disability (19). Thus, an early prediction of r-tPA effect is important for neurological physicians in emergency department. Although many studies have focused on the long-term prognosis of AIS after intravenous thrombolysis or biomarkers to predict intracranial hemorrhage, few studies focused on the r-tPA at a super-early stage (20, 21). In this study, we analyzed the clinical data of 339 patients and found a negative relationship of FDPs levels with r-tPA effect by using the NIHSS score at admission and 1 h after thrombolysis.

FDPs are fragments released by the plasmin-mediated degradation of fibrinogen or fibrin. These fragments can compete with fibrinogen for binding to the platelet membrane and interfere with platelet aggregation. Sun et al. reported that early assays of fibrinogen and FDP may be useful for predicting the risk of post-thrombolytic intracerebral hematoma (10). Nihei et al. found that high FDPs values may be a marker useful for differential diagnosis between patent-type acute aortic dissection and AIS (22). However, no study has focused on the relationship between FDPs levels and the thrombolysis effect of r-tPA, especially in the early time window. In our study, we found that patients' FDPs levels were negatively correlated with the thrombolysis effect, which was evaluated by NIHSS scores at admission and 1 h after r-tPA administration. To the best of our knowledge, this result has not been previously reported.

Monitoring representative markers with high specificity can provide a clearer view of alterations of a patient's fibrinolytic systems. Biomarkers such as D-dimer, uric acid, or homocysteine are most reported in practice for the diagnosis and early management of AIS. Chang et al. had reported that cystatin C is associated with unfavorable clinical outcomes after IV-tPA therapy in AIS at 90 days (23). As more than 60% of patients who receive rt-PA fail to recanalize, it is critical to find a biomarker to predict the r-tPA effect early in the treatment for better decision making in the emergency department. Plasma FDPs levels, which are an important index in coagulation function, is examined for every patient with AIS, and in this study we showed that FDP levels may be used as a biomarker for early evaluation of r-tPA thrombolysis.

Accurate prediction of the thrombolytic effect of r-tPA aids further treatment decisions and facilitates the effective use of limited medical resources. The NIHSS score is widely used to assess the severity, neurological deficits, and prognosis of patients with ischemic stroke (24). In our study, NIHSS scores were obtained at the time of admission and at 1 h after r-tPA administration. To gauge the efficacy of the intravenous r-tPA, an NIHSS score of ≤1 point 1 h after thrombolytic therapy or a decrease in the NIHSS score of ≥4 points after treatment compared with the NIHSS score at admission were considered favorable thrombolytic effects, while an NIHSS score improvement of <4 was considered an unfavorable effect (14).

In addition, a nomogram can facilitate management-related decision making by providing an individualized judgment, by assigning an appropriate weight to each variable based on its prognostic value and calculating a final score by combining independent variables (25). In our study, we employed a nomogram to further elucidate the factors that possibly influence the thrombolysis effect of r-tPA and display the probability factors for poor outcomes. FDPs, white blood cell count, age, D-dimer, and BMI were here found to increase the probability of adverse outcomes of r-tPA therapy. Most studies have reported predictive value for FDPs levels after r-tPA in cases of cerebral hemorrhage, whereas studies considering the effect of r-tPA 1 h after cerebral thrombolysis are scarce (26, 27).

Although our study provided some evidence for prediction of the thrombolytic effect of early use of r-tPA suitable for decision making for patients with AIS, it had some limitations that should not be ignored. First, we only used the NIHSS score at 1 h to evaluate the thrombolytic effect of r-tPA; the observation time should be extended to observe the long-term effect of early thrombolysis. Second, further validation and assessment of the applicability of the nomogram is required, requiring future prospective studies. Third, although the prognostic model can help clinicians accurately and effectively predict the prognosis, it cannot completely replace the judgments and diagnoses made by clinicians based on the individual characteristics of the patient. Finally, the sample size in this retrospective study was insufficient, which may have influenced the statistical significance of the logistic regression results.

## Conclusion

In summary, the FDPs levels at admission were negatively correlated with the r-tPA thrombolysis effect at the 1-h evaluation. AIS patients presenting with high FDP levels should receive more attention based on the NIHSS score 1 h after r-tPA administration. However, as sample size is quite small, the conclusion should be validated in a large cohort in the future.

## Data Availability Statement

The authors declared that all data underlying the findings are fully available which can be obtained after submitting a request to the hospital. Requests to access these datasets should be directed to Zhigang Zhao, 1022zzg@sina.com.

## Ethics Statement

The study was approved by the Ethics Committee of Beijing Tiantan Hospital and conducted in accordance with the Declaration of Helsinki. Due to the retrospective nature of the study, the requirement of written informed consent was waived. Confidential patient information was deleted from the entire data set prior to analysis.

## Author Contributions

XZ and ZZ conceived the presented idea. BZ wrote the manuscript. YT, WD, LZ, MW, TW, XL, and YL assisted with participant recruitment and data entry. JY assisted with data analysis and interpretation. All authors have read and approved the final version of the manuscript.

## Conflict of Interest

The authors declare that the research was conducted in the absence of any commercial or financial relationships that could be construed as a potential conflict of interest.
